# Impact of robotic-assisted surgery on length of hospital stay in Paris public hospitals: a retrospective analysis

**DOI:** 10.1007/s11701-024-02031-4

**Published:** 2024-09-04

**Authors:** Thomas Blanc, Carmen Capito, Edward Lambert, Pierre Mordant, François Audenet, Alexandre de la Taille, Matthieu Peycelon, Pierre Cattan, Jalal Assouad, Christophe Penna, Bruno Borghese, Morgan Roupret

**Affiliations:** 1 Department of Pediatric Surgery and Urology, Hôpital Necker-Enfants Malades, 149 rue de Sèvres, 75743, Paris Cedex 15, France; 2 Assistance Publique-Hôpitaux de Paris, Paris, France; 3 Université Paris Cité, Paris, France; 4 Sorbonne University, GRC 5 Predictive Onco-Uro, AP-HP, Urology, Pitie-Salpetriere Hospital, Paris, France; 5 J-ERUS/YAU Academic Urologists Working Group on Robot-Assisted Surgery, Paris, France; 6 Inserm, Physiopathologie et épidémiologie des maladies respiratoires, Paris, France; 7 Department of Vascular Surgery, Thoracic Surgery, and Lung Transplantation, Hôpital Bichat, INSERM 1152, Paris, France; 8 Department of Urology, Hôpital Européen Georges-Pompidou Hospital, Paris, France; 9 Department of Urology, Henri Mondor Hospital, University of Paris Est Créteil (UPEC), Créteil, France; 10 Department of Pediatric General Surgery and Urology, Robert-Debré University Hospital, National Reference Center for Rare Urinary Malformations (C.R.M.R. MARVU), Paris, France; 11 Digestive Surgery, Saint Louis Hospital, Paris, France; 12 Department of Thoracic Surgery, Tenon Hospital, Sorbonne University-Assistance Publique Hôpitaux de Paris, Paris, France; 13 Department of Digestive Surgery, APHP, Hôpital Bicêtre, Université Paris Saclay, Le Kremlin-Bicetre, France; 14 Service de Chirurgie Gynécologie Obstétrique II et Médecine de la Reproduction, Hôpital Universitaire Paris Centre (HUPC), Centre Hospitalier Universitaire (CHU) Cochin, Paris, France; 15 Genomics, Epigenetics and Physiopathology of Reproduction Team, Department of Development, Reproduction and Cancer, INSERM U1016, Paris, France

**Keywords:** Laparoscopy, Length of hospital stay, Open surgery, Robot-assisted surgery

## Abstract

The number of available hospital beds is decreasing in many countries. Reducing the length of hospital stay (LOS) and increasing bed turnover could improve patient flow. We evaluated whether robot-assisted surgery (RAS) had a beneficial impact on the LOS in a French hospital trust with a long-established robotic program (Assistance Publique–Hôpitaux de Paris, AP-HP). We extracted data from “Programme de Médicalisation des Systèmes d’Information” to determine the median LOS for adults in our trust after RAS versus laparoscopy and open surgery in 2021–2022 for eight target procedures, and compared data nationally and at similar academic centres (same database). We also calculated the number of hospitalisation days ‘saved’ using RAS. Overall, 9326 target procedures were performed at AP-HP: 3864 (41.4%) RAS, 2978 (31.9%) laparoscopies, and 2484 (26.6%) open surgeries. The median LOS for RAS was lower than laparoscopy and open surgery for all procedures, apart from hysterectomy and colectomy (equivalent to laparoscopy). Results for urological procedures at AP-HP reflected national values. The equivalent of 5390 hospitalisation days was saved in 2021–2022 using RAS instead of open surgery or laparoscopy at AP-HP; of these, 86% represented hospitalisation days saved using RAS in urological procedures. Using RAS instead of open surgery or laparoscopy (particularly in urological procedures) reduced the median LOS and may save thousands of hospitalisation days every year. This should help to increase patient turnover and facilitate patient flow.

**Supplementary Information:**

The online version contains supplementary material available at 10.1007/s11701-024-02031-4.

## Introduction

The number of hospital beds has fallen in many countries over the past few decades [1]. Although low bed numbers may demonstrate efficient patient care and a faster return to home, they can also lead to high bed occupancy rates. This makes it difficult to find beds for patients and affects patient flow, increasing the already heavy burden on a dwindling supply of nurses [2].

One solution is to reduce the length of hospital stay (LOS), i.e., the number of days that an inpatient will remain in hospital. This measure is often used to improve patient care, appropriately allocate resources where needed, and reduce overall costs [3]. A long hospital stay can lead to patient complications (e.g., infections or falls that could further prolong the LOS) and higher costs [4]. Reducing hospital stay and bed occupancy while maintaining optimum patient care should help to improve patient flow and lead to more efficient operational planning and performance [5].

Many factors can affect how long a patient needs to stay in hospital [6, 7]. In surgical patients, the type of procedure used has an impact on hospital stay; using minimally invasive surgery where possible instead of open surgery significantly reduces LOS [8–10], with robotic-assisted surgery (RAS) leading to a further reduction [11–16]. A robotic program has been long established at the Assistance Publique–Hôpitaux de Paris (AP-HP), a network of 39 University Hospitals operating in and around Paris, France, and the largest hospital system in Europe. A robotic system was first installed in 1999 at Broussais Hospital and moved to Georges Pompidou European Hospital for cardiac procedures, followed by two more robotic systems in 2000 (Mondor Hospital) and 2004 (La Pitié-Salpêtrière Hospital), which were mainly dedicated to urological procedures. In 2019, nine additional systems were bought. Since then, 13 robotic systems have been in use at AP-HP, allowing 2200 RAS procedures to be performed in 2021 and 2250 in 2022.

Our long robotic experience using standardised procedures within different surgical specialties makes us ideal to evaluate the impact that RAS has had on LOS within our hospital trust. Therefore, we aimed to use a French national database to determine the median LOS after RAS, laparoscopy, and open surgery in AP-HP and compare the data with values nationally and at similar academic centres, using the same database. We also calculated the estimated number of hospitalisation days that we were able to ‘save’ using RAS. Our hypothesis was that RAS has had a beneficial impact on LOS within AP-HP, and that using RAS would reduce the number of hospitalisation days compared with open surgery.

## Methods

This was a retrospective analysis of data obtained from the prospective French national database “Programme de Médicalisation des Systèmes d’Information” (PMSI). All patients signed informed consent to authorize prospective data collection and retrospective data analysis. The database was declared to the National Board for Informatics and Freedom (Commission Nationale Informatique et Liberté, CNIL, authorization #2217817) and to the National Institute for Health Data (Institut National des Données de Santé, INDS, authorization #MR1313290420). The PMSI is used to comprehensively record data for every surgery in all French hospitals. Each surgical procedure is encoded according to the specific Classification Commune des Actes Médicaux (CCAM) classification. In mid-2019, codes for RAS were created for urological, gynaecological, colorectal, and thoracic procedures, thus enabling surgical activity to be measured and traced within each hospital and at the national level.

In our analysis, all robotic surgeries performed at AP-HP hospitals were included and compared to those at the national level and at similar French academic hospitals (presumably with similar patient profiles to AP-HP). There were no exclusions, apart from surgeries that were performed at paediatric hospitals (which carry out 200 paediatric RAS per year). As RAS codes were not exhaustive for 2019, and because surgical activity was impacted by COVID-19 in 2020, only data for 2021 and 2022 were retrieved. The procedures for which RAS were used (and for which there was sufficient volume to allow analysis) were radical prostatectomy, partial and total nephrectomy, benign and malignant hysterectomy, rectal resection, colectomy, and pulmonary lobectomy. For the purposes of this article, we were only granted access to selected data within the PMSI (such as diagnoses, surgical procedure, LOS [17]). Thus, the outcomes extracted from the PMSI were a) the use of RAS compared with laparoscopy or open surgery for each procedure at AP-HP in relation to the French national average, and b) the median (interquartile range, IQR) LOS for each surgical technique at AP-HP versus the national median and the median at other academic hospitals. As the PMSI is a medico-administrative database, not a clinical evidence database, no cleaning methods were applied. No person-level information was used and there is no data linkage with other databases. For further information, please refer to the Technical Agency for Information on Hospitalization [17].

We then estimated the theoretical number of hospitalisation days that had been saved using RAS at AP-HP. To do this, we used the PMSI 2021–2022 data to obtain the average proportion of open and laparoscopic surgeries performed in non-equipped hospitals at the national level. We applied this percentage to the number of RAS performed at AP-HP in 2021–2022 to estimate the number of laparoscopic and open surgeries that could have taken place if RAS had not been used at AP-HP. Next, we applied the median LOS for laparoscopy and open surgery in non-equipped hospitals at the national level (PMSI 2021–2022) to this estimated volume of laparoscopic and open surgeries at AP-HP. This enabled us to determine the theoretical number of hospitalisation days that would have been recorded if AP-HP had not used RAS, and all patients had instead been treated with laparoscopy or open surgery. From this value, we subtracted the number of hospitalisation days actually recorded after RAS at AP-HP (PMSI 2021–2022), to give an estimated number of hospitalisation days ‘saved’ using RAS at AP-HP.

Data were extracted from PMSI 2021–2022 for eight hospitals (11 robotic systems) within AP-HP that have an RAS program, which does not include the two paediatric hospitals (Necker-Enfants Malades Hospital and Robert-Debré Hospital). Results below are highlighted for urological procedures, as the corresponding robotic program within AP-HP is larger and longer established than in other departments. The higher number of robotic urological surgeries performed (beyond average usage in France) will provide a greater insight into the impact of RAS at AP-HP. To give an overall perspective, results are also provided for all of the other RAS indications already listed for which there was a sufficient volume to allow analysis. All data can be made available upon reasonable request to the corresponding author.

## Results

### Use of RAS at AP-HP versus the national average

As illustrated in Table 1, 9326 target procedures were performed at AP-HP in 2021–2022: 3864 (41.4%) RAS, 2978 (31.9%) laparoscopies, and 2484 (26.6%) open surgeries. Compared to national values, the rate of minimally invasive surgery (RAS and laparoscopy) was higher at AP-HP for prostatectomy, partial nephrectomy, and benign and malign hysterectomy, while the rate of RAS alone was higher for all procedures but particularly for prostatectomy, partial and total nephrectomy, and benign and malignant hysterectomy.

**Table 1:** Use of RAS compared with open surgery and laparoscopy at AP-HP in 2021–2022 (source: PMSI 2021–2022)

	AP-HP							National data		
	Total	RAS		Open surgery		Laparoscopies		RAS	Open surgery	Laparoscopies
	*N*	*n*	%	*n*	%	*n*	%	%	%	%
Prostatectomy	1377	1171	**85.0**	71	5.2	135	9.8	58.5	18.4	23.1
Nephrectomy										
Partial	1037	780	**75.2**	192	18.5	65	6.3	56.3	24.0	19.7
Total	1030	448	**43.5**	327	31.7	255	24.8	19.4	29.7	50.9
Hysterectomy										
Benign	1601	533	**33.3**	354	22.1	714	44.6	4.9	30.4	64.7
Malign	592	333	**56.2**	100	16.9	159	26.9	11.3	29.0	59.7
Pulmonary lobectomy	1621	333	**20.5**	818	50.5	470	29.0	12.3	41.6	46.1
Rectal resections	818	143	**17.5**	280	34.2	395	48.3	9.2	24.3	66.5
Colectomies	1250	123	**9.8**	342	27.4	785	62.8	4.9	26.8	68.3
Total	9 326	3 864	41.4	2 484	26.6	2 978	31.9	**–**	**–**	**–**

Values in bold indicate the surgery(s) where the proportion (%) of procedures performed using RAS at AP-HP is higher than the national value

*AP-HP* Assistance Publique–Hôpitaux de Paris, *PMSI* Programme de Médicalisation des Systèmes d’Information, *RAS* robotic-assisted surgery

### Median LOS at AP-HP versus the national value and other academic centres

Online Resource 1 shows that the median LOS for urological procedures using RAS at AP-HP was lower than both laparoscopy (by 1 day each for prostatectomy, partial nephrectomy, and total nephrectomy) and open surgery (by up to 4 days for prostatectomy, 2 days for partial nephrectomy, and 3 days for total nephrectomy). This reflects the trend observed nationally. However, while the median LOS at other academic centres was always lower with RAS than open surgery, it was otherwise comparable between RAS and laparoscopy.

In other indications at AP-HP, the median LOS was lower for RAS than open surgery for all procedures, but was only lower than laparoscopy for pulmonary lobectomy and rectal resections. Nationally and at other academic centres, the median LOS was always lower with RAS than open surgery, but values were otherwise generally similar to laparoscopy.

### Hospitalisation days saved

Overall, the equivalent of 5390 hospitalisation days were saved in 2021–2022 using RAS instead of open surgery or laparoscopy at AP-HP (Table 2). In particular, 2150 days were saved for prostatectomy, 1630 days for partial nephrectomy, and 840 days for total nephrectomy (i.e. 86% in urological procedures). The number of hospitalisation days saved in the other indications was lower due to the lower number of procedures performed, ranging from 0 days for pulmonary lobectomy to 370 days for benign hysterectomy.

**Table 2:** Estimated number of hospitalisation days saved within each indication using RAS at AP-HP (source: PMSI 2021–2022)^*a*^

Indication	Hospitalisation days saved with RAS, *n*
Prostatectomy	2 150
Nephrectomy	
Partial	1 630
Total	840
Hysterectomy	
Benign	370
Malignant	120
Pulmonary lobectomy	0
Rectal resections	150
Colectomies	130
Total	5 390

^a^See Methods section for calculation

*AP-HP* Assistance Publique–Hôpitaux de Paris, *PMSI* Programme de Médicalisation des Systèmes d’Information, *RAS* robotic-assisted surgery

## Discussion

The length of a hospital stay is an important indicator of how efficiently hospitals are managed, and has an impact on bed turnover. In addition to direct benefits for patients (including lower risk for readmission and mortality) [18], reducing the LOS should free up beds for new admissions and increase patient turnover—allowing more patients to be treated efficiently. In France, the average length of hospital stay (LOS) in 2019 was 8.8 days, which had decreased from 10.5 days in 2009 but was still higher than many other countries [19].

Minimally invasive surgeries such as laparoscopy or robotic surgery require smaller incisions, involve less trauma and blood loss, and result in faster recovery than traditional open surgery. Many meta-analyses have demonstrated that minimally invasive surgery significantly reduces LOS compared with open surgery [8–10]. It has also been shown that further reductions in LOS can be made using robotic surgery instead of laparoscopic surgery [11–16]. However, LOS varies considerably among patients with different diagnoses; even within the same diagnosis, LOS can be affected by factors, such as comorbidity, surgeon expertise, institutional volume, and differences in treatment complexity [3, 11].

We decided to analyse data within our own hospital trust (which has a long-established, high-volume robotic program and a higher rate of RAS than the French national average), using outcomes extracted from a prospective, national database of surgical outcomes (PMSI). Data for our largest robotic program in urology showed that the median LOS for such procedures in 2021–2022 (the first full years since codes for RAS were created) was always shorter after RAS than open *and* laparoscopic surgery. Comparison with the median LOS at the national level (using the same database and timeframe) confirmed that using RAS instead of laparoscopy or open surgery reduces hospital stay after urological procedures within France. These findings are consistent with those reported in the literature [11–13].

Analysis of the other types of procedures performed at AP-HP demonstrated that the median LOS was always lower with RAS than open surgery. When compared to laparoscopy, the median LOS was lower with RAS when used for pulmonary lobectomy and rectal resections and comparable when used for benign and malignant hysterectomy and colectomy. These results are encouraging, because RAS activity only began 3–4 years ago within some of these departments. At both the national level and at other academic centres, using RAS for these other indications always resulted in a lower median LOS than open surgery; when compared to laparoscopy, the median LOS was similar to RAS across these indications. It should be noted that the comparison of LOS following pulmonary lobectomy is mitigated by the fact that pulmonary lobectomy and segmentectomy have the same PMSI code, introducing a classification bias in the analysis.

We calculated that the reductions in the median LOS seen at AP-HP in 2021–2022 would have led to the equivalent of 5390 days of hospitalisation ‘saved’ by using RAS across all indications, including 4620 days saved for urological procedures alone. These savings could potentially enable surgical volume to be increased without the need for additional nurses. Furthermore, although we did not conduct a cost analysis, the reduction in hospital stay should translate into reduced costs and a more cost-efficient service [20].

A strength of our study is that a large number of hospitals (eight hospitals with 11 robots) from a single trust were included in the analysis. Although we have greater experience in robotic surgery compared with some hospitals, the comparison with national values and similar academic centres gives a better overall perspective of the impact of robotic surgery on LOS in France and suggests that our results could be generalised to less experienced hospitals. However, it was noticeable that the median LOS after laparoscopy and/or open surgery was sometimes longer in AP-HP than the averages nationally and in other academic hospitals (e.g., for malignant hysterectomy, lobectomy). This could be the result of a shortage of postoperative facility care in Paris or the lack of coordinating nurses in most hospitals of our public network. The observation may be of importance, because although LOS was substantially reduced using RAS at AP-HP, the impact in other hospitals might not be as apparent. Furthermore, LOS is not just influenced by the surgical approach, but also by the level of perioperative care (e.g., implementation of enhanced recovery after surgery protocols). It is imperative that such information is included in any future data analysis. Even so, any reduction in LOS is to be welcomed. It is possible that an earlier discharge from hospital would have an impact on both the rate of readmission and the associated LOS after readmission. It is a limitation of our study that we do not yet have the readmission data for analysis, and we plan to follow-up with further morbidity, mortality, and readmission data when available. Previous research suggests, however, that there may be a lower risk of readmission in patients with a shorter LOS [18, 21]. In fact, the main limitation of our analysis was that the PMSI database did not include important parameters linked to disease characteristics and functional outcomes, which may affect the intraoperative process, intra- and postoperative complications, recovery times, etc. We were limited to analysis of the type of surgical approach and LOS. Clinical outcomes are also important drivers of health economics. A cost analysis would have been beneficial to verify the economic benefits of using RAS instead of laparoscopy or open surgery. In addition, only 2021–2022 data were included (the most recent years for which enough data were available), taken from a single database. The scope needs to be widened, and analysis of registry data will be performed after this initial analysis, including a closer look at patient characteristics that might have an impact on outcomes.

## Conclusion

Results from the AP-HP—a hospital network that uses robotic surgery at a higher rate for many common procedures than the national average—indicated that using RAS instead of laparoscopy or open surgery reduced the median length of hospital stay and potentially saved thousands of hospitalisation days every year. Ensuring patients receive optimum care while reducing the time they need to recover in hospital will enable more beneficial outcomes in patients and could increase patient turnover without the need for additional resources.

## Supplementary Information

Below is the link to the electronic supplementary material.

Supplementary file1 (PDF 62 KB)

## Data Availability

All data can be made available upon reasonable request to the corresponding author.
