# Supplementary material for: Impact of robotic-assisted surgery on length of hospital stay in Paris public hospitals: a retrospective analysis
Source: J Robot Surg. 2024 Sep 4;18(1):332. doi: 10.1007/s11701-024-02031-4 (PMC11374824; doi:10.1007/s11701-024-02031-4)
Supplement: Supplementary file 1 — Supplementary file1 (PDF 62 KB) [file 11701_2024_2031_MOESM1_ESM.pdf]

Impact of robotic-assisted surgery on length of hospital stay in Paris public hospitals: a retrospective analysis

Thomas Blanc[1,2,3], Carmen Capito[1,2], Edward Lambert[2,4,5], Pierre Mordant[2,3,6,7], François Audenet[2,3,8], Alexandre de la Taille[2,9], Matthieu Peycelon[2,3,10], Pierre Cattan[2,3,11], Jalal Assouad[2,12], Christophe Penna[2,13], Bruno Borghese[2, 3,14,15], Morgan Roupret[4]

[1] Department of Pediatric Surgery and Urology, Hôpital Necker-Enfants Malades, Paris, France.

[2] Assistance Publique-Hôpitaux de Paris, Paris, France.

[3] Université Paris Cité, Paris, France.

[4] Sorbonne University, GRC 5 Predictive Onco-Uro, AP-HP, Urology, Pitie-Salpetriere Hospital, Paris, France.

[5] J-ERUS/YAU Academic Urologists Working Group on Robot-Assisted Surgery.

[6] Inserm, Physiopathologie et épidémiologie des maladies respiratoires, Paris, France.

[7] Department of Vascular Surgery, Thoracic Surgery, and Lung Transplantation, Hôpital Bichat, INSERM 1152, Paris, France.

[8] Department of Urology, Hôpital Européen Georges-Pompidou Hospital, Paris, France.

[9] Department of Urology, Henri Mondor Hospital, University of Paris Est Créteil (UPEC), Créteil, France.

[10] Department of Pediatric General Surgery and Urology, Robert-Debré University Hospital, National Reference Center for Rare Urinary Malformations (C.R.M.R. MARVU), Paris, France.

[11] Digestive Surgery, Saint Louis Hospital, Paris, France.

[12] Department of Thoracic Surgery, Tenon Hospital, Sorbonne University-Assistance Publique Hôpitaux de Paris, Paris, France.

[13] Department of Digestive Surgery, APHP, Hôpital Bicêtre, Université Paris Saclay, Le Kremlin-Bicetre, France.

[14] Service de Chirurgie Gynécologie Obstétrique II et Médecine de la Reproduction, Hôpital Universitaire Paris Centre (HUPC), Centre Hospitalier Universitaire (CHU) Cochin, Paris France

[15] Genomics, Epigenetics and Physiopathology of Reproduction Team, Department of Development, Reproduction and Cancer, INSERM U1016, Paris, France.

Corresponding author. Prof. Thomas Blanc, Department of Pediatric Surgery and Urology, Hôpital Necker-Enfants Malades, Assistance Publique-Hôpitaux de Paris, 149 rue de Sèvres, 75743 Paris Cedex 15, France. **ORCID ID: 0000-0002-5829-8167**.

Supplementary Table S1. Overall median (interquartile range) LOS for each procedure and type of surgery within each indication for 2021 and 2022. Highlighted cells indicate the surgery(s) that resulted in the lowest LOS compared to the other two surgeries within each dataset (source: PMSI 2021-2022).

	AP-HP			National data			Other academic centres		
	**RAS**	**Laparoscopy**	**Open**	**RAS**	**Laparoscopy**	**Open**	**RAS**	**Laparoscopy**	**Open**
**Prostatectomy**									
2021	3.0 (2.0)	4.0 (1.0)	7.0 (1.0)	3.0 (2.0)	4.0 (4.0)	7.0 (4.0)	3.0 (2.0)	4.0 (1.0)	5.0 (4.0)
2022	3.0 (1.0)	4.0 (2.0)	6.5 (3.0)	3.0 (2.0)	4.0 (4.0)	6.0 (4.0)	3.0 (2.0)	3.0 (1.0)	4.0 (3.0)
**Nephrectomy**									
**Partial**									
2021	3.0 (2.0)	4.0 (2.0)	5.0 (3.0)	3.0 (3.0)	4.0 (2.0)	6.0 (3.0)	3.0 (2.0)	3.0 (2.0)	6.0 (3.0)
2022	3.0 (2.0)	4.0 (2.75)	5.0 (3.0)	3.0 (2.0)	4.0 (3.0)	6.0 (4.0)	3.0 (2.0)	3.0 (2.0)	5.0 (3.0)
**Total**									
2021	4.0 (3.0)	5.0 (3.0)	7.0 (7.0)	4.0 (3.0)	5.0 (3.0)	7.0 (5.0)	4.0 (3.0)	4.0 (3.0)	7.0 (8.0)
2022	4.0 (3.0)	5.0 (3.0)	7.0 (7.0)	4.0 (3.0)	5.0 (4.0)	8.0 (7.0)	4.0 (3.0)	4.0 (3.0)	8.0 (9.0)
**Hysterectomy**									
**Benign**									
2021	2.0 (2.0)	2.0 (2.0)	4.0 (2.0)	2.0 (2.0)	2.0 (2.0)	4.0 (2.0)	2.0 (2.0)	2.0 (2.0)	4.0 (2.0)
2022	2.0 (2.0)	2.0 (2.0)	4.0 (2.0)	2.0 (2.0)	2.0 (1.0)	4.0 (2.0)	2.0 (2.0)	2.0 (2.0)	4.0 (2.0)
**Malignant**									
2021	3.0 (2.0)	3.0 (2.0)	7.0 (5.0)	3.0 (2.0)	2.0 (2.0)	6.0 (3.0)	3.0 (2.0)	3.0 (2.0)	6.0 (5.0)
2022	3.0 (2.0)	3.0 (2.0)	7.0 (6.0)	2.0 (2.0)	2.0 (2.0)	6.0 (4.0)	2.0 (2.0)	3.0 (1.5)	6.0 (4.0)
**Pulmonary lobectomy**									
2021	6.0 (4.0)	7.0 (4.0)	8.0 (6.0)	6.0 (4.0)	5.0 (4.0)	7.0 (5.0)	6.0 (4.0)	5.0 (4.0)	7.0 (5.0)
2022	6.0 (4.0)	7.0 (5.0)	8.0 (6.0)	5.0 (4.0)	5.0 (4.0)	7.0 (5.0)	5.0 (4.0)	6.0 (4.0)	7.0 (5.0)
**Rectal resections**									
2021	8.0 (4.25)	8.0 (7.0)	11.0 (10.0)	8.0 (6.0)	8.0 (6.0)	12.0 (9.0)	8.0 (6.0)	8.0 (6.0)	12.0 (11.0)
2022	8.0 (4.25)	9.0 (6.0)	12.0 (12.0)	8.0 (6.0)	8.0 (6.0)	12.0 (10.0)	8.0 (5.0)	8.0 (7.0)	12.0 (12.0)
**Colectomies**									
2021	6.0 (2.0)	6.0 (3.0)	10.0 (9.0)	6.0 (3.0)	6.0 (4.0)	10.0 (7.0)	6.0 (3.0)	6.0 (4.0)	11.0 (9.0)
2022	6.0 (4.0)	6.0 (3.0)	10.0 (9.0)	6.0 (5.0)	6.0 (4.0)	10.0 (9.0)	6.0 (4.0)	6.0 (3.0)	11.0 (10.0)

Abbreviations: AP-HP, Assistance Publique–Hôpitaux de Paris; LOS, length of stay; PMSI, Programme de Médicalisation des Systèmes d'Information; RAS, robotic-assisted surgery.
